# Modeling the Mutualistic Interactions between Tubeworms and Microbial Consortia

**DOI:** 10.1371/journal.pbio.0030077

**Published:** 2005-02-22

**Authors:** Erik E Cordes, Michael A Arthur, Katriona Shea, Rolf S Arvidson, Charles R Fisher

**Affiliations:** **1**Biology Department, Pennsylvania State UniversityUniversity Park, PennsylvaniaUnited States of America; **2**Geosciences Department, Pennsylvania State UniversityUniversity Park, PennsylvaniaUnited States of America; **3**Department of Earth Science, Rice UniversityHouston, TexasUnited States of America; Monterey Bay Aquarium Research InstituteUnited States of America

## Abstract

The deep-sea vestimentiferan tubeworm Lamellibrachia luymesi forms large aggregations at hydrocarbon seeps in the Gulf of Mexico that may persist for over 250 y. Here, we present the results of a diagenetic model in which tubeworm aggregation persistence is achieved through augmentation of the supply of sulfate to hydrocarbon seep sediments. In the model, L. luymesi releases the sulfate generated by its internal, chemoautotrophic, sulfide-oxidizing symbionts through posterior root-like extensions of its body. The sulfate fuels sulfate reduction, commonly coupled to anaerobic methane oxidation and hydrocarbon degradation by bacterial–archaeal consortia. If sulfate is released by the tubeworms, sulfide generation mainly by hydrocarbon degradation is sufficient to support moderate-sized aggregations of L. luymesi for hundreds of years. The results of this model expand our concept of the potential benefits derived from complex interspecific relationships, in this case involving members of all three domains of life.

## Abstract 2

Modeling the interactions between deep-sea tubeworms and bacteria/archaea at hydrocarbon seeps provides a solution to their long term energy source and could help to explain the tubeworm's extreme longevity

## Introduction

Complex positive species interactions have been shown to expand the ecological niche and increase the persistence of the organisms involved in a variety of systems. In terrestrial systems, increased diversity of mycorrhizal symbionts is correlated with increased biodiversity of plant communities, resulting in greater stability and longer persistence at the community level [1]. In marine ecosystems, the coral Oculina arbuscula harbors a majid crab, *Mithrax forceps,* that prevents overgrowth of macroalgae and shading of the corals [2]. This allows O. arbuscula to maintain its facultative mutualism with photosynthetic zooxanthellae in well-lit habitats off the Atlantic coast of North Carolina, increasing the amount of energy available to the coral for growth and reproduction. At cold seeps in the Cascadia [3,4] and Aleutian [5] subduction zones, bioirrigation through burrow formation and bioturbation by clams (*Calyptogena* spp.) has been shown to significantly affect the distribution of microbial anaerobic methane oxidation.

Lamellibrachia luymesi inhabits areas associated with advection of hydrocarbons and other reduced chemicals to the seafloor (hydrocarbon or brine seeps) on the upper Louisiana slope (ULS) of the Gulf of Mexico from 400 to 1,000 m depth. L. luymesi does not posses a digestive system; rather, it acquires energy via internal sulfide-oxidizing bacterial symbionts [6]. L. luymesi differs from other vestimentiferan tubeworms by its ability to use a posterior extension of its body, the “root,” to acquire sulfide from interstitial pools in sediments [7,8]. Near the anterior plumes of tubeworms, sulfide concentrations typically decline below 0.1 μM as the tubeworms approach 1 m in length [9]. By using its roots, L. luymesi is able to delve into deeper sediment layers, providing access to more persistent sulfide sources. In the apparent absence of lethal predation [10,11], the most significant hazard that this vestimentiferan tubeworm faces is sulfide limitation. Its high uptake rate of sulfide from hydrocarbon seep sediments, estimated at over 30 μmol · h^−1^ for a moderate-sized individual [12], suggests that sulfide flux may be limiting in L. luymesi's habitat.

A diverse chemosynthetic community relies on the sulfide generated as a by-product of anaerobic degradative processes in the Gulf of Mexico [10,11]. Reduction of seawater sulfate utilizing methane or other hydrocarbons as electron donors produces the majority of sulfide available at ULS seeps [13,14]. Anaerobic methane oxidation is most commonly carried out by microbial consortia consisting of sulfate-reducing bacteria along with methanogenic archaea executing reverse methanogenesis [15,16]. Methane oxidation linked to sulfate reduction and subsequent authigenic carbonate precipitation constrain ocean–atmosphere carbon fluxes [3,4], accounting for up to 20% of the global methane flux to the atmosphere [17]. Oxidation of other hydrocarbons and organic material, carried out by sulfate-reducing bacteria in monoculture and in consortia with other microbes [18], may account for a larger proportion of sulfate depletion in ULS sediments [14]. These processes can result in a decoupling of sulfate reduction and methane oxidation rates [14], and form carbonates consisting mainly of non-methane-derived carbon [19]. L. luymesi may influence these anaerobic processes by utilizing its roots to release the sulfate generated by its symbionts during sulfide oxidation [7,8,12]. This hypothetical mechanism would provide sulfate for anaerobic methane oxidation and hydrocarbon degradation at sediment depths normally devoid of energetically favorable oxidants, thereby augmenting exogenous sulfide production.

In this study, we address the question of whether known biogeochemical processes could supply sulfide at rates sufficient to match the requirements of long-lived L. luymesi aggregations. In the diagenetic model presented here, the hypothesized release of sulfate in sediments with sufficient electron donors results in sulfide generation at rates matching the sulfide uptake rate of L. luymesi aggregations for over 250 y. We speculate that the mutual benefits derived from the syntrophy among symbiotic tubeworms and microbial consortia implicit in the model would expand our current concept for the potential complexity of positive interspecific interactions and the benefits they confer.

## Results/Discussion

### L. luymesi Sulfate Release Allows Persistence of Aggregations

The model predicts that inputs from known sources, including diffusion and advection of deep sulfide along with reduced seawater sulfate, will support a moderately-sized aggregation of 1,000 individuals for an average of 39 y (range, 22 to 78 y) (Figure 1). A smaller aggregation of 200 individuals could be maintained with these sources for an average of 64.1 y (standard deviation, 10.6 y). In this model configuration, the duration of adequate sulfide flux is not congruent with the known sizes of aggregations and existing age estimates of L. luymesi individuals and aggregations. Adding sulfate release by tubeworm roots to the model results in sulfide generation and flux at rates that match the demands of large aggregations, allowing the tubeworms to survive for over 250 y (Figure 1). This additional source of sulfate results in a two-orders-of-magnitude increase in sulfate flux in older (>100 y) aggregations, accounting for over 90% of sulfate available after only 24 y. The sulfate released by the tubeworms would be used for anaerobic methane oxidation and hydrocarbon degradation. The nature of the relationship between symbiotic tubeworms and microbial consortia that we are proposing is a coupling of the sulfur cycle only, and not carbon. Light dissolved inorganic carbon (DIC) resulting from the oxidation of hydrocarbons is apparently not taken up by tubeworms as the carbon stable isotope signatures of L. luymesi are heavier than would be expected from a methane-derived DIC source [20,21]. In addition, the well-studied hydrothermal vent tubeworm, *Riftia pachyptila,* obtains carbon in the form of CO_2_ across its plume [22]. However, this does not necessarily exclude the passive diffusion of DIC across the root surface, which could account for some of the variability observed in L. luymesi carbon stable isotope signatures [20,21]. By augmenting the sulfate supply to microbial consortia for sulfate reduction, large aggregations of tubeworms may survive for hundreds of years in the model, mirroring the population sizes and individual lengths regularly observed and collected at seeps on the ULS [23].

**Figure 1:**
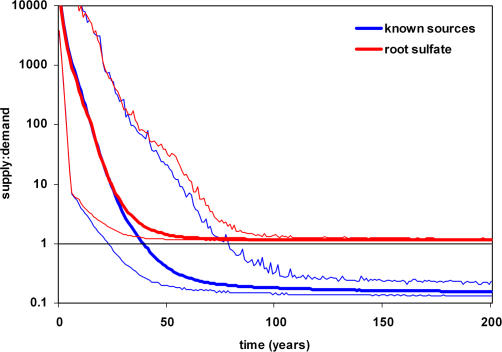
Ratio of Sulfide Supply to Sulfide Uptake Rate of L. luymesi Aggregations Equilibrium line (1:1 ratio) and average, maximum, and minimum values for 1,000 iterations presented. Supply rate based on known sources without sulfate release by tubeworm roots shown in blue. Sulfide supply declines below demand after approximately 40 y. Supply rate including sulfate release from tubeworm roots shown in red, with sulfate release constrained by tubeworm symbionts' sulfide oxidation rate. Sulfide supply exceeds demand for the duration of the model.

### Model Results Are Robust to Parameter Variation

An alternate hypothesis to explain the discordance between estimated sulfide supply and uptake rates is the presence of locally elevated seepage rates. Sensitivity analyses were carried out to determine the potential effects of uncertainty in seepage rate on supply estimated for aggregations without root sulfate release. A 10% increase in seepage rate resulted in a 5.6% increase in sulfide supply to aggregations 200 y old and older. This corresponds to only 16.4% of the sulfide required, which does not serve to extend aggregation survivorship (average, 39 y; range, 21 to 79 y) beyond that determined for lower flow rates. To supply the sulfide flux required by older aggregations, seepage rate would have to be at least 363 mm · y^−1^. This is over ten times greater than the rate used in the model (32 mm · y^−1^), which is the highest region-wide estimate for the Gulf of Mexico [24]. A rate of over 300 mm · y^−1^ approaches rates reported for active venting of fluids (Table 1). Active venting would result in the visual manifestation of seepage in the form of methane bubbles and oil droplets, which are generally restricted to mussel (Bathymodiolus childressi) beds at these sites [25]. In addition, larger, and therefore older [26], aggregations have lower epibenthic sulfide concentrations [8,9,25] suggesting that seepage becomes less vigorous over time and is not in the form of active venting in larger tubeworm aggregations. While difficult to obtain, in situ measures of advection rate of fluids at Gulf of Mexico seeps could be used to test these assumptions and may lend insight into the relationship between variability in tubeworm growth rate and sulfide availability.

**Table 1:**
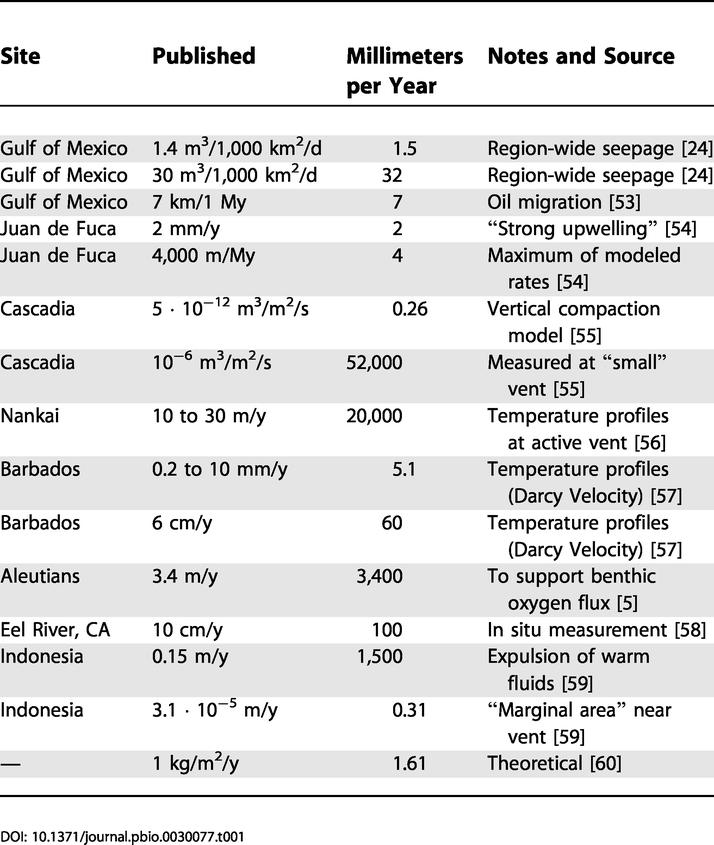
Reported Seepage Rates for Hydrocarbon and Methane Seeps

The high degree of variability in growth rate and recruitment rate could also affect the ratio of supply and demand in the model. In an aggregation exhibiting anomalously low recruitment, the size of the rhizosphere would increase more rapidly than the biomass of the aggregation. This would lead to high rates of sulfide delivery and generation and low rates of sulfide uptake by tubeworm roots. When initial recruitment rate (*a* in equations 1 and 2) is decreased by 10%, the length of time that supply exceeds demand increases by 3.7%. This effect appears to be linear, with a 20% decrease in initial recruitment rate resulting in a 7.4% increase in persistence. If growth rate is increased, thereby increasing the rate of rhizosphere growth in terms of surface area for diffusion and advection, there appears to be little effect of the ratio of supply to demand (20% increase in growth—0% change in persistence time). In fact, increasing growth to the upper limits of the error term (equation 5) lowers the amount of time that the aggregation can be supported since biomass and sulfide demand increase more rapidly than increases in supply resulting from additional surface area. By decreasing growth rate, aggregations may be supported for longer periods of time, with a 20% decrease leading to a 6.3% increase in persistence time and a decrease of 88% leading to persistence for over 250 y. While an 88% lower growth rate lies outside of the range of existing growth data, this could be accomplished by ceasing growth for extended periods of time in a quiescent stage. This possibility remains to be investigated in L. luymesi. By utilizing a variable recruitment rate in the model, both between realized aggregations and between years within a model run, along with a growth error term encompassing the full range of observed growth data, the model is capable of generating aggregations within the range of the 10%–20% variability tested in this analysis. Even these outlying aggregations (presented as maxima and minima in Figure 1) support the qualitative conclusions drawn from model results.

While the model was based on empirical data to the greatest degree possible, estimates of many of the parameters necessary to resolve the model were not available or are extremely difficult to measure in deep water with existing technology. Uptake rates were measured in the laboratory [8] for relatively small individuals (<50 cm). While we attempted to approximate metabolic scaling by covarying uptake and growth rates, it is possible that large individuals require even lower sulfide flux. Model predictions are not overly sensitive to variability in this parameter. A reduction by 10% of the overall sulfide uptake rate results in a 5.2% increase in persistence time. To maintain an aggregation for over 250 y, mass-specific uptake rate would have to be reduced 6-fold. While this could also be accomplished by entering a period of quiescence as mentioned before, there is no existing evidence for this ability in vestimentiferans.

The second version of the model is based on the assumption that L. luymesi is capable of releasing sulfate through its roots. It should be noted that in the model, sulfate release is constrained by the rate of sulfate generation by the tubeworm's sulfide-oxidizing symbionts, resulting in the near 1:1 ratio of supply and demand in Figure 1. Though modeled sulfate flux across the roots into the rhizosphere may exceed 20 mmol · h^−1^ in older aggregations, the roots provide an ample respiratory surface such that rates of sulfate flux per unit root surface area do not exceed 0.4 μmol · h^−1^· cm^−2^ in the model. It remains possible that a proportion of the sulfate could be released through the plume of the tubeworms, though the energy required to pump sulfate against a concentration gradient (seawater [SO_4_] = 29 mM) [13] suggests that it would be more energetically favorable for the sulfate to passively diffuse out of the roots. It is also possible that sulfate flux could be increased by active bioirrigation delivering seawater to deeper sediment layers through the tubeworm tubes. This could allow the sulfide-oxidizing symbionts to store some of the oxidized sulfide as elemental sulfur rather than releasing it as sulfate, while maintaining sufficient sulfate flux to deeper sediment layers for sulfide generation. These mechanisms remain hypothetical and require further experimental investigations to evaluate their potential role in this system.

### Tubeworms Impact Seep Biogeochemistry

Tubeworm sulfate release, in conjunction with high sulfide uptake rates, could contribute to the observation of declining advection rate in older aggregations. By increasing sulfate flux to deeper sediments, L. luymesi increases integrated rates of anaerobic methane oxidation and hydrocarbon degradation, which would enhance authigenic calcium carbonate precipitation within the rhizosphere. Under the conditions of root sulfate release in the model, calcium carbonate precipitation is rapid (0.109 to 0.316 μmol · l^−1^ · sec^−1^) in the first 53 y, with rates declining exponentially thereafter. By creating a barrier to fluid advection [4], this could result in the observed decrease in epibenthic sulfide concentration in older aggregations [8,9] and the predicted cessation of tubeworm recruitment around this time [12,23].

In order to prevent the precipitation of carbonate directly on the root surface, L. luymesi individuals may release hydrogen ions as well as sulfate through their roots. While hydrogen ion flux through the roots has not yet been empirically demonstrated, none of the nearly 5,000 tubeworms examined as part of this study were observed to have carbonate formed directly on their roots, suggesting that this form of precipitation is inhibited in some manner. In the model, diffusion of hydrogen ions across the root surface (the only form of release explicitly modeled) accounts for less than 40% of ion generation when carbonate precipitation is most vigorous. We speculate that L. luymesi may utilize the excess hydrogen ions generated by their sulfide-oxidizing symbionts to periodically raise the rate of hydrogen ion flux from their roots. This would not only supply additional hydrogen ions to sulfate-reducing bacteria, but could inhibit carbonate precipitation on the tubes and subsequent reduction of the root area utilizable as a respiratory surface. Further pH reduction could dissolve existing carbonate in sediments beneath the rhizosphere, thereby opening seepage pathways and allowing further root growth. This possibility is corroborated by the observation of young tubeworms that had apparently bored through bivalve shells in an experimental system (R. Carney, personal communication). Empirical measurements of hydrogen ion flux across the root tissue of L. luymesi are required to test these hypothetical mechanisms.

The release of sulfate by tubeworm roots potentially explains the frequent observation of highly degraded hydrocarbons in the vicinity of large tubeworm aggregations [27]. The majority of sulfate supplied by tubeworm roots is utilized for microbial hydrocarbon degradation in the model (Figure2). This process alone accounts for over 60% of the sulfide available to aggregations after approximately 80 y. In the absence of liquid and solid phase hydrocarbons, methane flux would have to be approximately four times the rate in the model in order to fuel sufficient sulfate reduction to support an aggregation for over 200 y. This could occur in sediments overlying rapidly sublimating gas hydrates, and hydrate abundance has been previously suggested as a potential factor influencing the distribution of chemosynthetic communities in the Gulf of Mexico [10]. However, model results indicate that large chain hydrocarbons are the most significant energy source for sulfate reduction in tubeworm-dominated sediments. Increased integrated rates of hydrocarbon degradation would lead to highly biologically altered hydrocarbon pools among the roots of tubeworm aggregations. Hydrocarbon oxidation has been implicated as one of the dominant processes in the carbon cycle at ULS seeps, accounting for over 90% of the carbon in carbonates collected in the vicinity of tubeworm aggregations [19]. Model analysis indicates that the minimum amount of organic carbon (including hydrocarbons as well as buried organic material) in sediments required to supply sulfide at rates matching aggregation demand (1:1 supply:uptake ratio) is 1.03% by weight, remarkably close to the lowest value found in any of the seep sediment core samples (1.2%) [13,28], and greater than that found in ULS sediments away from seeps (0.71%) [29]. Determination of organic carbon concentration in sediments beneath tubeworm aggregations is necessary to test the prediction that elevated carbon content at seeps, primarily resulting from oil seepage, provides the energy source required to generate sufficient sulfide for tubeworm aggregations.

**Figure 2:**
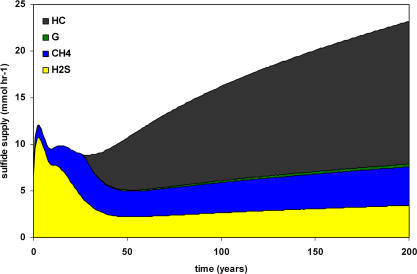
Sources of Sulfide Available to Tubeworm Aggregations over Time in the Model Sources of sulfide include advection and diffusion of sulfide from deep sources (yellow) or sulfate reduction using methane (blue), buried organic carbon (green), or C_6+_ hydrocarbons (dark grey) as electron donors. Sulfate is provided by diffusion from sediments surrounding the rhizosphere, diffusion at the sediment–water interface, and release from tubeworm roots.

Additional sulfate flux from tubeworm roots could also explain the high apparent sulfate diffusion coefficients determined for tubeworm-impacted sediments [13]. Anomalous sulfate fluxes have been proposed to be a result of bioturbation and bioirrigation by macrofauna [3,5], and recycling by microbial mats [13]. The results of the model presented here provide evidence for macrofaunal sulfur recycling, an additional component to be considered in future investigations of cold seep biogeochemistry. The hypothesized release of sulfate by tubeworm roots potentially explains numerous, apparently disparate observations, hinting at the great impact that L. luymesi aggregations may have on their abiotic environment.

While the proposed interactions between symbiotic tubeworms and sulfate-reducing bacteria are essential for the persistence of L. luymesi aggregations in the model, we suggest that there are significant effects on the microbial community as well. This syntrophy will increase the abundance of sulfate-reducing bacteria and therefore increase the rates of anaerobic methane oxidation and hydrocarbon degradation carried out by microbial consortia that rely on sulfate as an oxidant. Tubeworm-generated sulfate supplies a more energetically favorable electron acceptor below the normal depth of sulfate penetration at seeps, relaxing the limitation on anaerobic oxidative processes at these sediment depths. Deeper sediment layers then become habitable to sulfate reducers, significantly altering the microbial community structure within the rhizosphere. Model configurations neglect the potential role of bioirrigation of seawater sulfate through L. luymesi tubes, which could further increase sulfate supply to deeper sediment layers. The possible role of tubeworm roots as substrata for the growth of microbial consortia, analogous to the habitat afforded mycorrhizal symbionts of higher plants, remains another possible benefit for the microbes. These predictions may be tested by determination of the relative abundance of microbial consortia at different depths of sediments both impacted by and isolated from tubeworms. Localization of the microbes on the root surface would provide evidence for a more intricate relationship. It is our hope that the results of this model may provide the impetus for future rigorous experimental tests of these ideas.

### Summary

The model results presented here are consistent with the hypothesis that L. luymesi releases sulfate into hydrocarbon-rich sediments to fuel sulfide generation, allowing for the persistence of the longest-lived animal known. The importance of this process to sulfide generation in the modeled rhizosphere implies a complex relationship between an animal with bacterial endosymbionts and external sulfate-reducing bacteria, often in consortia with methane-oxidizing or hydrocarbon-degrading microbes. This positive interspecific relationship, including members of all three domains, would benefit both the tubeworms and the microbial consortia involved. This expands our existing concept of the potential for complexity in mutualisms and the benefits they may confer. Further complex relationships are likely to be discovered through continued research into the role of positive species interactions at the individual and community levels.

## Materials and Methods

### 

This study couples an individual-based population growth and sulfide uptake model [12] to a diagenetic diffusion/advection model to compare the relative magnitude of sulfide supply and uptake for long-lived tubeworm aggregations. A series of 1,000 iterations of the model under three different initial conditions (known sources of sulfate, known sources plus root sulfate supply, and known sources with elevated seepage rates) were carried out. The rhizosphere (volume of sediment encompassed by the root system of an aggregation) is modeled as an inverted dome beneath the sediment with a radius equal to the average root length of the population (Figure 3). The rhizosphere was approximated by a series of two-dimensional discs at 2-cm intervals in order to reduce the complexity of a three-dimensional solution for a sphere of changing size. Sulfate (SO_4_^2−^), methane (CH_4_), sulfide (HS^−^), bicarbonate (HCO_3_^−^), and hydrogen ion (H^+^) fluxes across the rhizosphere boundary are determined. Sulfate reduction rates using methane, larger chain hydrocarbons, and buried organic matter as electron donors are modeled in order to estimate the sulfide available to tubeworm aggregations as they change in size over the course of 250 y.

**Figure 3:**
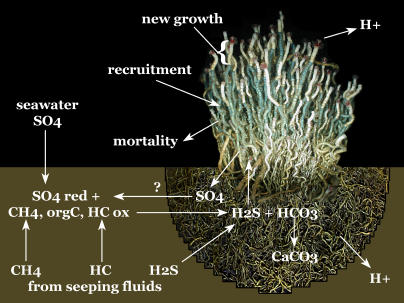
Model Construction Population model includes individual size-specific growth and mortality rates, and population size-specific recruitment rate. Growth rate was determined by in situ staining of tubeworm aggregations using a blue chitin stain (in situ photograph of stained aggregation demonstrating annual growth shown here) and collection after 12–14 mo. Diagenetic model included advection and diffusion of sulfate, sulfide, methane, bicarbonate, and hydrogen ions as well as organic carbon content of sediments. Fluxes across the rhizosphere (root system) boundary were compared to sulfide uptake rates for simulated aggregations to determine whether sulfide supply could match the required uptake rates of aggregations (for specific methodology see methods). HC, C_6+_ hydrocarbons; orgC, organic carbon; ox, oxidation reaction; red, reduction reaction.

#### Population growth model

The population growth model follows the methodology presented in [12] and includes population growth, mortality rate, individual growth rate, and sulfide uptake rate. The parameters underlying the population growth model were refined using growth data from an additional 615 individuals and population data from an additional 11 aggregations comprising 4,908 individuals. The model presented here includes data from a total of 23 tubeworm aggregations from three nearby sites (Green Canyon oil lease blocks 184, 232, and 234) collected over a period of 7 y on the ULS to arrive at generalized population growth parameters.

L. luymesi individuals are dioecious, with males releasing sperm into the water column. Fertilization is believed to be external [30], though sperm has been found within the oviducts of females of the hydrothermal vent tubeworm R. pachyptila [31]. Eggs and embryos are positively buoyant and develop into a swimming trochophore-like larval stage within 3 d of fertilization [32]. Larvae are lecithotrophic and may remain in the water column for several weeks [32]. They require hard substrata for settlement, and acquire symbionts from their environment after metamorphosis [33,34]. Settlement is initially rapid, and continues until the available substrate is occupied [12,23,35]. Population sizes of aggregations collected with existing sampling devices typically vary between 100 and 1,500 individuals ([12,23]; this study), though far larger aggregations covering tens to hundreds of square meters are common at the sites sampled. Previous studies have shown that L. luymesi has an average longevity of 135 y [12], and requires an average of 210 y to reach 2 m in length [26], a size not uncommon among collected animals. Mortality events are exceedingly rare, dropping below 1% annual mortality probability for animals over 30 cm [12]. The expanded datasets of growth and mortality rates included here extend the longevity estimate for L. luymesi to an average of 176 y and the estimated age of a 2-m-long animal to 216 y.

At the beginning of each iteration, population growth parameters are chosen for the following population growth model:



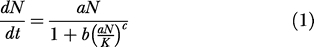



where *N* is population size, *t* is time (in years), *K* is carrying capacity (set to 1,000 individuals for all simulations presented here), *a* describes the initial slope of the line, *b* defines the degree of density dependence, and *c* is a shape parameter. The first parameter *(a)* was generated using the following function:



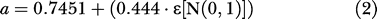



where *ɛ*[N(0,1)] is a normally distributed random deviate with an average of zero and a standard deviation of one. This allows the initial recruitment rate to vary within the range of all recruitment trajectories that have been observed [12]. The other parameters were not normally distributed; therefore, the log-transformed distributions were used to define the distribution of the random numbers generated. As the three parameters in the model were significantly correlated (ln(*a*) and ln(*b*), *r* = −0.853, *p* < 0.001; ln(*a*) and ln (*c*), *r* = −0.461, *p* = 0.036), values of *b* and *c* were chosen from their relationship with *a*:



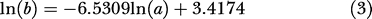





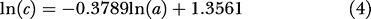



The value of *a* was allowed to vary each year according to the pooled standard error associated with the estimates of *a* from the empirical data (standard error, 0.105). Once population size equaled or exceeded carrying capacity, recruitment was ceased, representing the lack of additional substrate or sulfide available in the water column.

Once recruitment was determined for that year, the individual-based portion of the model began. Each individual was traced through time with respect to its length, root length, mass, mortality probability, mass-specific sulfide uptake rate, sulfate excretion rate, and hydrogen ion elimination rate. Growth rates of tubeworms were determined by staining tubes in situ (Figure 3) and collection 12 to 14 mo later ([26]; this study). Individual growth rate was determined from the following function (Figure 4):

**Figure 4:**
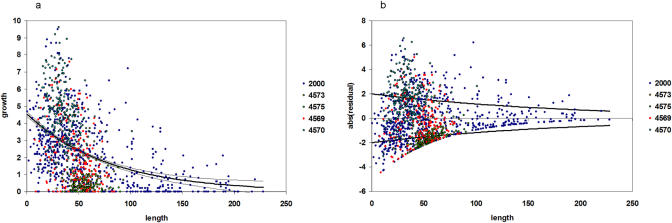
L. luymesi Growth Rate Size-specific growth of L. luymesi determined from stained tubeworms. Different colors indicate growth data from different aggregations. Blue points labeled “2000” are all from Bergquist et al. [26]. Other colored points refer to submersible dive numbers from 2003 when stained aggregations were collected. (A) Growth function and 95% confidence interval for size-specific growth. (B) Error function fitted to the residuals of the model.



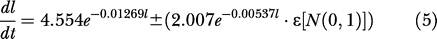



Length *(l)* is defined here as the distance from the anterior end of the tube to an outer tube diameter of 2 mm following the methodology of [26]. All growth rates were standardized to 365 d. The error term is an additional function fitted to the residuals of the first regression function (Figure 4B), resulting in a variable growth rate. This error term was used rather than varying growth within the 95% confidence interval of the regression of length and growth rate because of the high degree of variability in growth among individuals. It should also be noted that there is a certain degree of variability in growth rate between aggregations (Figure 4). This may be attributable to spatial or temporal variability in seepage rate or sulfide concentration between aggregations. Aggregations may be subject to persistently differing conditions on a small (meter) scale, or may encounter periodic fluctuations in habitat characteristics. Because we are uncertain whether this variation is persistent on the temporal scales that we are simulating, between-aggregation variability is not explicitly modeled, though by chance certain realized aggregations deviated from mean growth rate.

The ratio of root length to tube length was determined from individual length using the following function:



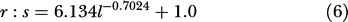



Annual mortality rate was approximated as the size-specific frequency of empty tubes in collected aggregations [12] with an overall annual mortality rate of 0.569%. This approximation is conservative and likely overestimates yearly mortality, as available data indicate that empty tubes should persist longer than 1 y [12,36]. Mortality probability was determined for each 10-cm size class using the following function:



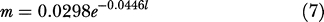



where *m* is mortality probability and *l* is length. Individuals were considered dead if their probability of mortality exceeded a uniform random number between zero and one.

By using generalized population growth parameters in the model presented here, we attempt to encompass the range of empirical data from sampled aggregations in our examination of sulfide uptake and supply rates. Taken together, the population growth model including recruitment, growth, and mortality provides a good qualitative if not quantitative fit for any individual aggregation, reflecting the size frequency of tubeworms within sampled aggregations [12]. It should be noted that the modeling of specific aggregations was not the aim of this study; rather, an attempt has been made to encompass the variability observed in the various populations of tubeworms that have been sampled. To examine the effect of uncertainty in the population growth parameters, sensitivity analyses were carried out. The initial slope of the recruitment rate (*a* in equation 1) was varied while individual size-specific growth rate was held constant (no error term in equation 5). Growth rate was then varied while holding the initial rate of population growth constant (no error term in equation 2). The effect of a 10% change in each parameter was determined, and then changes of greater magnitude were examined to determine the fastest rate of population or individual growth that could be supported by the sulfide available to the aggregation in the absence of sulfate release.

Individual sulfide uptake was allowed to vary within the range of laboratory-determined sulfide uptake rates according to that individual's growth rate for that year:



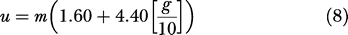



where *u* is uptake rate (in micromoles per gram per hour), *m* is mass (in grams), and *g* is growth rate (in centimeters per year). Growth rate was divided by the maximum growth rate (10 cm · y^−1^) such that highest growth rates resulted in highest uptake rates. By scaling uptake rate with growth, we approximate metabolic scaling, resulting in a decline in uptake rate by a factor of 3.7 over the range of tubeworm sizes in this study [12]. The amount of sulfate that could be excreted by each individual was determined from the amount generated by sulfide oxidation carried out by the internal chemoautotrophic symbionts assuming constant internal sulfate concentration, thereby accounting for changes in body volume. We do not account for the binding of sulfur by free amino acids, as this is believed to relatively minor compared the flux rates of sulfate and sulfide, and is reversible [37]. Hydrogen ions are also generated in the oxidation of sulfide by the tubeworm symbionts. Hydrogen ion elimination rate was determined in the model in the same fashion as sulfide uptake, with growth rate determining the variability in this metabolic flux according to laboratory-measured ion fluxes (mean, 10.96 μmol · g^−1^ · h^−1^; standard deviation, 1.88 μmol · g^−1^ · h^−1^) [38]. Simple diffusion of hydrogen ions across the root surface was included in the model, though the exact mode of proton flux has not yet been determined experimentally for L. luymesi [38]. As diffusion across the roots accounts for a relatively small proportion of total proton flux (less than 10% in large individuals), additional pathways are likely and require further investigation.

#### Geochemical setting

Known sources of sulfide available to L. luymesi aggregations are sulfide transported with seeping fluids [10] and sulfide generated via reduction of seawater sulfate [39,40]. The majority of the sulfide present at ULS sites is believed to be related to sulfate reduction coupled to anaerobic hydrocarbon oxidation [14,39]. Other potential sources of sulfide associated with seepage include anaerobic oxidation of deeply buried organic material [10], “sour” hydrocarbons containing a proportion of sulfur [41], and hydrocarbon interactions with sulfur-bearing minerals such as gypsum and anhydrite found in the salt dome cap rocks of the ULS [8,42,43].

Concentrations of all chemical species in the sediments surrounding the rhizosphere were derived from the dataset included in Arvidson et al. [13] and Morse et al. [28]. Only those sediment cores taken around the “drip line” of tubeworm aggregations that contained detectable sulfide concentrations were used. Due to the vagaries of sampling with a submersible in sediments heavily impacted by carbonate and roots, those cores with detectable sulfide are believed to more accurately represent conditions around the periphery of the rhizosphere.

Dissolved organic carbon (DOC) concentration was used as an estimate of methane concentration. While other forms of DOC make up this total concentration, methane accounts for 90%–95% of the hydrocarbon gasses dissolved in pore waters [28]. In seep sediments, the majority of DOC is likely to be in the form of hydrocarbon gasses. Because estimates of organic acid concentrations were not available, they could not be explicitly modeled. This would not affect the overall concentration of electron donors in the model, but could affect the sulfate reduction rate. Since sulfate reduction rate estimates for methane seeps in the Gulf of Mexico are among the highest recorded [14,39], any differences in DOC composition (e.g., higher relative concentrations of dissolved organic acids) would serve to lower the overall sulfate reduction rate and sulfide availability. Sulfide supply estimates presented are likely overestimated most by the model without root sulfate release owing to the greater reliance on anaerobic methane oxidation in this form of the model. Simulations including sulfate release by tubeworms are affected to a lesser extent as the concentration of electron donors is not limiting in this model configuration.

Solid and liquid phase organic carbon was separated into hydrocarbons and buried organic material according to their relative concentrations in hydrocarbon seep and surrounding Gulf of Mexico sediments. Background sediments on the ULS contain 0.71% organic carbon by weight [29]. At hydrocarbon seeps on the ULS, organic carbon accounts for 4.47% of total weight. This was assumed to be the sum of background organic input plus carbon in the form of C_6+_ hydrocarbons. It is possible that the higher biomass located at ULS seeps in the form of non-living macrofaunal and microbial materials may also contribute to the increased organic carbon concentration, but without empirical estimates, this could not be accounted for in the model. Hydrocarbons may consist of between 50% and 95% labile materials [44,45,46]. Based on existing data on degradation rates and residual hydrocarbons subjected to degradation [42,47], a value of 50% labile material was used here. These assumptions of hydrocarbon concentration and degradation potential are therefore believed to be conservative.

The following functions were fitted to the sulfide, sulfate, and methane concentration profiles (Figure 5) to determine the boundary conditions at any given depth:

**Figure 5:**
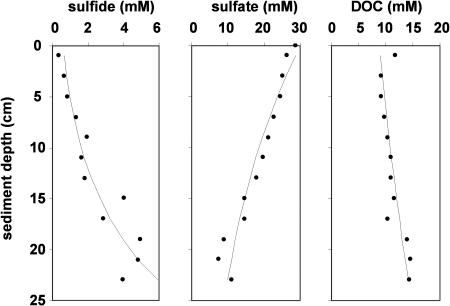
Concentration Profiles of Sulfate, Sulfide, and DOC Points represent average concentration at a given depth from 13 sediment cores taken around the periphery of tubeworm aggregations (see Materials and Methods and original data in [13,28]). Best-fitted line based on least squares fit of equation 9.



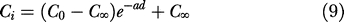



where *C*_0_ is initial concentration, *C_∞_* is concentration at infinite distance, and *C_i_* is concentration at depth *d*. As there were no existing data for sediments below 30 cm, concentrations at infinite depth (*C_∞_*) were used (SO_4_^2−^ = 0 mmol · l^−1^, HS^−^ = 12 mmol · l^−1^, DOC = 11 mmol · l^−1^, DIC = 20 mmol · l^−1^, pH = 7.78). The first derivatives of the sulfide and methane profiles were used for the calculation of advective flux from depth. The first derivative of the sulfate profile was used for diffusive flux across the water–sediment interface of the rhizosphere, with advection rate subtracted from diffusive flux of sulfate across this surface. Advection (seepage) rate varied with time according to the following function:



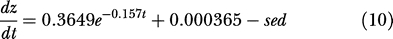



where *t* is simulation time in years and *sed* is sedimentation rate (6 cm · 1,000 y^−1^) [29]. Early seepage rate approximated the highest flux rates measured or estimated for methane seeps and declined with time in the model to the highest estimates for persistent, region-wide seepage in the Gulf of Mexico (Table 1). This follows a pattern of hydrocarbon seep development, with the highest seepage rates early in the evolution of the local seepage source followed by occlusion of fluid migration pathways by carbonate precipitation, hydrate formation, and possibly tubeworm root growth. By using the highest rate estimated (32 mm · y^−1^ = 0.000365 cm · h^−1^ in equation 10) as the basal seepage rate, we are testing the possibility that tubeworm aggregations could survive under the most favorable conditions possible in the absence of tubeworm sulfate supply.

For sediments encompassed by the rhizosphere, sulfide, sulfate, methane, DOC, and hydrogen ion concentration profiles were determined iteratively prior to model implementation using a central difference scheme:



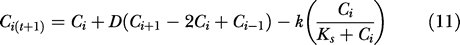



where *C*_*i*(*t*)_ is concentration in cell *i* at time *t, D* is the diffusion coefficient, *k* is the maximum reaction rate, and *K_s_* is the half-saturation constant for the reaction (Table 2). Reactions included anaerobic methane oxidation (equation 17), tubeworm sulfide uptake rate (equation 8), and carbonate precipitation rate (equation 22). The concentration in each 2 × 2 cm cell was calculated at 1 h time steps. At the end of each year, diffusion distance increased. The number of cells (total diffusion distance) was determined by the average root length of L. luymesi populations as realized in independent runs of the population growth model described above, and included here as model input only. A separate function was fitted to each of the concentration profiles:

**Table 2:**
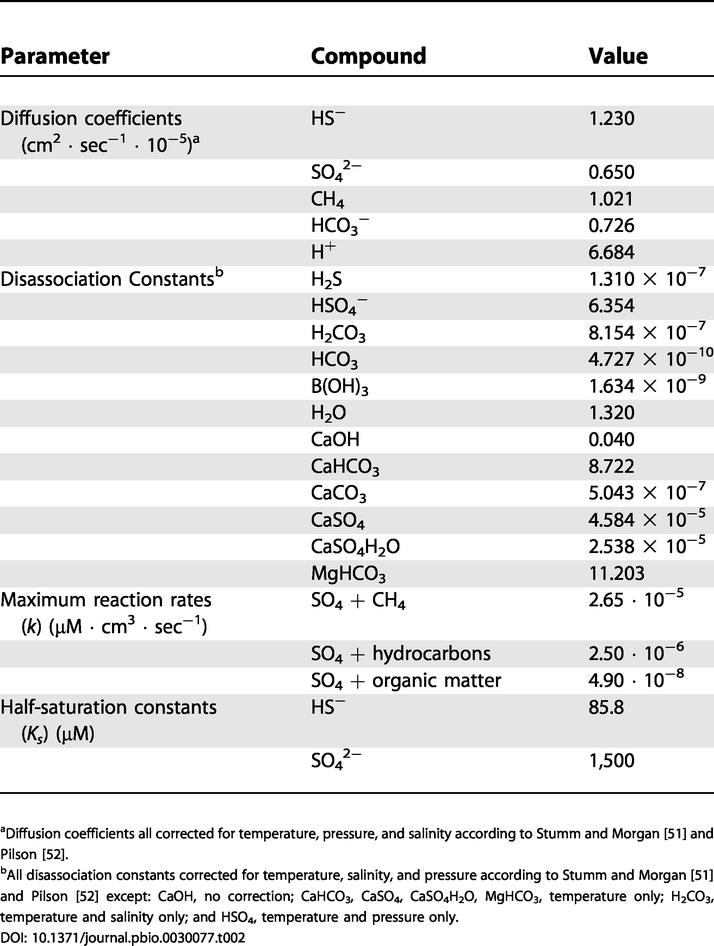
Parameters Involved in Diagenetic Model

^a^Diffusion coefficients all corrected for temperature, pressure, and salinity according to Stumm and Morgan [51] and Pilson [52]

^b^All disassociation constants corrected for temperature, salinity, and pressure according to Stumm and Morgan [51] and Pilson [52] except: CaOH, no correction; CaHCO_3_, CaSO_4_, CaSO_4_H_2_O, MgHCO_3_, temperature only; H_2_CO_3_, temperature and salinity only; and HSO_4_, temperature and pressure only



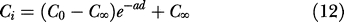



where *d* is radial distance. The relationship between the parameter *a* and distance was used to generate concentration profiles for each disc comprising the rhizosphere. Because of the tight linear relationship between diffusion distance and the shape of the curve, concentration profiles could be generated for a disc of any size using the following function:



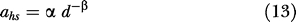



where α is 1.7344 and β is 1.0104 for HS^−^, α is 0.2111 and β is 0.3363 for SO_4_^2−^, and α is 0.1626 and β is 0.2518 for CH_4_. Diffusional fluxes of sulfide, sulfate, and methane were calculated according to the first and second derivatives of the concentration profiles as determined by the diameter of each disc.

#### Model implementation

The model estimates sulfide availability to the aggregation as a whole by summing the fluxes separately determined for each 2-cm disc composing the rhizosphere. Depth-dependant boundary conditions were set for each disc separately based on the sediment profiles (Figure 5). Diffusional fluxes into each disc were calculated from the shape of the concentration profiles according to the following function [48]:



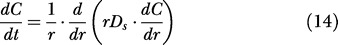



where *C* is concentration, *r* is disc radius, and *D_s_* is the diffusion coefficient corrected for porosity by:



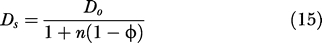



where *D_o_* is the diffusion coefficient corrected for temperature and pressure, *n* is the chemical species-specific constant, and φ is porosity. The value of *n* was set to 2.75 as this was found to be a reasonable fit for all chemical species examined [49]. The ionic states of each species at the average pH value of tubeworm-dominated sediments (7.78) were used for the determination of diffusion coefficients. Porosity was determined from the following function:



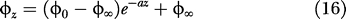



where φ_*z*_ is porosity at depth *z,* φ_0_ is porosity at the sediment–water interface, and φ_∞_ is porosity at infinite depth; φ_0_ was set at 0.841, φ_∞_ at 0.765, and *a* at 0.210, as determined from the best fit with the porosity data (Figure 6) from Morse et al. [28].

**Figure 6:**
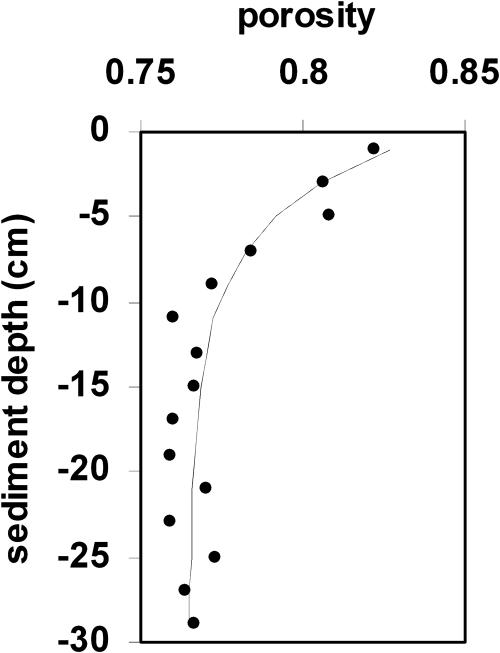
Sediment Porosity Values Points represent average porosity at a given depth from 13 sediment cores taken around the periphery of tubeworm aggregations (see Materials and Methods and original data in [13,28]). Best-fitted line based on least squares fit of equation 9.

Diffusion across the sediment–water interface of the rhizosphere was also considered as an additional input of sulfate and hydrogen ions. This was included as one-dimensional diffusion across a circular surface (subtracting the area encompassed by the tubeworm tubes) with diffusion distance equal to rhizosphere diameter, and concentration differential from seawater concentration to the average concentration within the rhizosphere. Sulfate and hydrogen ion diffusion across the root surface was then added (if included in the set of model realizations) as simple Fickian diffusion. Concentration differential was the difference between internal concentration and average concentration for each disc of the rhizosphere assuming roots were evenly proportioned according to the volume encompassed by each disc. Internal sulfate concentration and pH (Table 2) represented an average of the values determined for R. pachyptila [22], a hydrothermal vent tubeworm. Internal sulfate concentrations and pH of L. luymesi have not been reported, but these values are generally consistent within taxa [50]. Uptake of sulfide and release of sulfate were summed across the entire tubeworm population, again assuming an even distribution of roots within the rhizosphere. The paucity of empirical data on the location of any individual tubeworm's roots within an aggregation precluded modeling space explicitly; therefore, it is assumed that each individual has equal access to the resources available within the rhizosphere.

Within the rhizosphere, sulfide generation may be limited by sulfate supply, electron donor availability, or sulfate reduction rate. Sulfate supply was determined as the sum of flux across the series of discs approximating the rhizosphere dome, across the sediment–water interface, and from root sulfate (if available). Available sulfate is utilized for anaerobic methane oxidation first (the more energetically favorable process), then hydrocarbon and organic matter degradation. Electron donors included methane, complex hydrocarbons, and buried organic material. Solid and liquid phase hydrocarbons and organic material were assumed to be homogenous within the rhizosphere. Methane supply was determined as the sum of flux across each rhizosphere disc boundary. Hydrocarbon and organic material concentrations were determined as the amounts encompassed within the rhizosphere volume minus that oxidized in previous years. Sulfate reduction rate was determined from the relative amounts of the various electron donors with higher rates (0.71 μmol · ml^−1^ · h^−1^) for methane oxidation and lower rates (0.083 μmol · ml^−1^ · h^−1^) for organic matter or hydrocarbon degradation [39]. Microbes carrying out these processes are assumed to be evenly distributed within the rhizosphere.

Total hydrogen sulfide availability to the aggregation was determined as the sum of sulfide diffusion and advection across each rhizosphere disc and sulfide generated within the rhizosphere from sulfate reduction according to the following reactions:


SO_4_^2−^ + CH_4_ → HS^−^ + HCO_3_^−^ + H_2_O

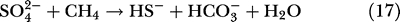




SO_4_^2−^ + 2CH_2_O → HS^−^ + 2HCO_3_^−^ + H_2_O

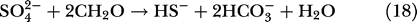




SO_4_^2−^ + 1.47C_n_H_2n+2_ → HS^−^ + 1.47HCO_3_^−^ + H_2_O

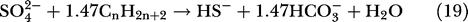



Bicarbonate (HCO_3_^−^) is generated at a 1:1 stoichiometry during anaerobic methane oxidation and a 2:1 stoichiometry in the degradation of organic material. As hydrocarbons are degraded forming smaller chain hydrocarbons and organic acids, bicarbonate is generated at different stoichiometries. Because different-sized hydrocarbons and organic acids were not accounted for in the model, a rough average of these stoichiometries (1.47:1) based on toluene, ethylbenzene, xylene, and hexadecane degradation [18] was used to determine the amount of bicarbonate generated per mole of carbon. Hydrogen ions are also used up in a 1:1 stoichiometry with sulfate in the sulfate reduction half reaction as included in reaction 17.

In order to account for carbonate precipitation, the model traced DIC concentration, calcium concentration, hydrogen ion concentration, buffer capacity, carbonate saturation, and carbonate precipitation rate. The buffer state of the rhizosphere was calculated to determine changes in pH resulting from hydrogen ion flux. Buffer capacity (*β*) was calculated using the following function [51]:



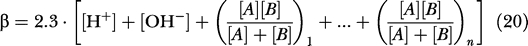



where *A* and *B* represent the concentrations of the various acids and bases in the buffer system. In addition to hydrogen and hydroxyl ions, the buffer system included carbonate (CO_2_, H_2_CO_3_, HCO_3_^−^, and CO_3_^2−^), sulfide (H_2_S and HS^−^), sulfate (HSO_4_^−^ and SO_4_^2−^), and borate (B[OH]_4_^−^ and B[OH]_3_) speciation. Current pH was used to determine the ionic state of each species according to temperature-, pressure-, and salinity-corrected disassociation constants when available [51,52] (Table 2). Change in pH was determined from hydrogen ion flux and buffer capacity as follows:



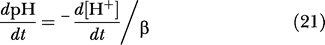



Saturation state is highly dependent on the degree to which calcium and bicarbonate form complexes with other ions. The “free” calcium was determined as the proportion of calcium that is not associated with complexed bicarbonate (HCO_3_^−^), carbonate (CO_3_^2−^), hydroxyl (OH^−^), or sulfate (SO_4_^2−^) ions. Free carbonate was determined as the amount not forming complexes with calcium (Ca^+^) or magnesium (Mg^+^) ions in solution. Saturation state was then calculated from the product of the concentrations of free calcium and carbonate divided by the solubility product constant. If the saturation state was above one, then carbonate precipitation occurred at a rate determined by:



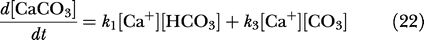



where *k*_1_ is 0.00597 l · mol^−1^ · sec^−1^ and *k_3_* = 0.456 l · mol^−1^ · sec^−1^ [51]. Because there is no empirical relationship between weight percent of carbonate and sediment porosity in tubeworm-dominated sediments [28], precipitation did not directly affect porosity. Precipitation was accounted for in the model by subtracting the volume of carbonate precipitate from the total volume encompassed by the rhizosphere.

At the end of each annual time step, model output included average length of individuals, population size, sulfide uptake rate, sulfide supply rate, root sulfate flux (if included), root hydrogen ion flux, amount of sulfide supply accounted for by each process (sulfide seepage, anaerobic methane oxidation, organic matter degradation, and hydrocarbon degradation), number of individuals that could be supported by sulfide supply, carbonate precipitation rate, volume of carbonate precipitate, and pH.

## Abbreviations

DIC: dissolved inorganic carbon
DOC: dissolved organic carbon ULS
